# Resistance, Tolerance, Virulence and Bacterial Pathogen Fitness—Current State and Envisioned Solutions for the Near Future

**DOI:** 10.3390/pathogens12050746

**Published:** 2023-05-22

**Authors:** Veronica Lazar, Eliza Oprea, Lia-Mara Ditu

**Affiliations:** Department of Botany and Microbiology, Faculty of Biology, University of Bucharest, 1-3 Portocalelor Street, 060101 Bucharest, Romania; veronica.lazar@bio.unibuc.ro (V.L.); lia-mara.ditu@bio.unibuc.ro (L.-M.D.)

**Keywords:** antibiotic resistance (AR) and multidrug-resistant, bacterial virulence and fitness, quorum sensing (QS) mechanism, biofilms, tolerance of biofilm cells, new antimicrobials, antipathogenic strategies

## Abstract

The current antibiotic crisis and the global phenomena of bacterial resistance, inherited and non-inherited, and tolerance—associated with biofilm formation—are prompting dire predictions of a post-antibiotic era in the near future. These predictions refer to increases in morbidity and mortality rates as a consequence of infections with multidrug-resistant or pandrug-resistant microbial strains. In this context, we aimed to highlight the current status of the antibiotic resistance phenomenon and the significance of bacterial virulence properties/fitness for human health and to review the main strategies alternative or complementary to antibiotic therapy, some of them being already clinically applied or in clinical trials, others only foreseen and in the research phase.

## 1. Introduction: Antibiotic Resistance, Where Are We Now?

One of the global priority health problems is antimicrobial resistance (AMR), which determines the high morbidity and mortality rates shown by superbugs—resistant and virulent bacterial strains—with an influence on the duration of hospitalization and costs for specific pathogen–drug combinations. The high level of AMR, the resistance gene pool (clinical and environmental reservoirs), and environmental pollution, especially by xenobiotics, including antibiotics which are considered water micropollutants, are among the most important problems the world is facing today [1].

Despite the great scientific progress in vaccination and chemotherapy, infectious diseases remain a serious health issue, being among the leading causes of morbidity worldwide and a top priority for public health. However, little progress has been made in the development of new antimicrobial drugs. Moreover, the wide use of antibiotics has evolutionary and ecological effects, leading to the recruitment of more genes into the resistome and mobilome, with negative consequences for human welfare and the environment too [2,3,4].

A systematic and comprehensive analysis, based on 471 million individual records (from 204 countries and territories) and using statistical methods, estimated that 4.95 million deaths were associated with bacterial resistance in 2019, including 1.27 million deaths directly due to bacterial AMR. Concerning localization, the lower respiratory infections caused by AMR strains were the cause of more than 1.5 million deaths correlated with AMR. For instance, a common pathogen, methicillin-resistant *Staphylococcus aureus* (MRSA), caused more than 100,000 deaths in 2019, and other common pathogens each caused between 50,000 and 100,000 deaths [5]. Six of the leading pathogens accounting for the global AMR in 2019—*E. coli*, *S. aureus*, *Klebsiella pneumoniae*, *Streptococcus pneumoniae*, *Acinetobacter baumannii* and *Pseudomonas aeruginosa*—were identified by the WHO as priority pathogens, which required a concerted combat plan, including surveillance, research work to discover new antibiotics or alternative/complementary antimicrobials/drugs and new vaccines. Speaking about this last alternative, only the pneumococcal vaccination is globally successful [6].

Antibiotic resistance (AR) of clinically important bacteria is rising in both community and hospital settings, with high mortality and morbidity rates. A committee of experts (*Eurosurveillence* program) estimated that by 2050, about 10 million people will die every year in the world from bacterial infections, exceeding cancer mortality [7].

There are three main types of AR: (i) innate or intrinsic resistance; (ii) acquired resistance, a consequence of selective pressure of antibiotics from the environment; and (iii) adaptative resistance that is due to the ecological niche conditions and includes genetic changes induced by the environment [8]. One of the most important mechanisms of the spread of AR is horizontal gene transfer (HGT). So, the acquired resistance is propagated between the community members by intraspecies, interspecies or intergenera HGT of mobile genetic elements (plasmids, transposons, integrons, phage genes) or by the acquisition of extracellular DNA fragments [9,10].

Among the most clinically relevant pathogens are those included in the group called ESKAPE, including *Enterococcus faecium*, *Staphylococcus aureus*, *Klebsiella pneumoniae*, *Acinetobacter baumannii*, *Pseudomonas aeruginosa* and *Enterobacter* spp. All of them present a high level of AR, express many virulence factors and are biofilm formers, being responsible for the increasing incidence of MDR infections in immunodeficient patients [11]. The major concern regarding these *opportunistic pathogens* is their great adaptability, correlated with many virulence factors, resistance mechanisms and fitness. So, there is an urgent and increased need to develop new strategies for preventing or managing infections with MDR and virulent strains.

From a biological point of view, AMR is recently evolved, during the last eight decades, after the beginning of antibiotic availability and intensive use. In fact, the phenomenon is natural and more ancient, being present in microbial antibiotic producers and in associated species in an ecological niche, compared with the recent accelerated evolution and spread of resistance genes. Antibiotics—these old substances, secondary metabolites with new functions as anti-infectious drugs—have made a major contribution to the reduction in morbidity and mortality rates due to infectious diseases in the world. However, the price was an adverse effect, a continuously increased antibiotic resistance (AR), favored by clinical and non-clinical excessive use or misuse of antibiotics and other antimicrobials [12].

The previous and actual data explain the necessity of WHO health programs at the global level and surveillance programs generating data that are essential to making adequate and local specific policy decisions, mainly for prevention and control programs, including access of low-resource regions/countries to essential antibiotics [5]. Even though these directions were established years ago, there are still gaps, and it is necessary to develop microbiology laboratory capacity and data collection systems to better understand the AMR development and spread phenomena, in order to discover and implement efficient counter methods.

For this article, our aim was to underline the actual level and significance of associated bacterial resistance and virulence properties/fitness and to review the main strategies alternative or complementary to antibiotics, some of them being already clinically applied or in clinical trials, others only foreseen and in the research phase.

## 2. Biofilms/Tolerance of Biofilm Cells Derived from the Social Behavior

One of the four crucial problems of public health, according to the European Center for Disease Control (ECDC), is represented by antibiotic resistance (AR). As has been shown, the excessive and uncontrollable use of antibiotics and other antimicrobials increased bacterial resistance, causing serious problems at a global level [13,14]. However, this problem is amplified when bacteria grow in biofilms.

Biofilms are bacterial/microbial communities formed when the free, floating or planktonic cells sediment and adhere to a surface, represented by inert or cellular substrates. So, a biofilm is a microbial mono- or polyspecific (in this case, a consortium) community with cells irreversibly attached to a substratum or an interface, embedded in a self-produced matrix and with a different phenotype concerning the growth rate, gene transcription and behavior [15].

This other state of existence of bacteria/microorganisms is based on a complex communication mechanism between cells, the quorum sensing (QS) and response system, which controls different processes, including the biofilm formation and the different behavior of component cells, which are more resistant to antibiotics and to host defense mechanisms by comparison with the free cells [14,16,17]. Some authors consider that the terms biofilm and antibiotic resistance (AR) are practically synonymous due to the biofilm behavioral resistance or tolerance to multiple antibiotics, a biofilm being at the same time the perfect niche for the exchange of resistance and/or virulence genes between the biofilm component cells [18]. This is explainable because, for clinicians and their patients too, the results of the phenomena mentioned above are the same: therapeutic failure and the correlated consequences (at an individual level, as well as at medical and socioeconomic levels).

Even though bacteria are, by definition, unicellular, the free-living or planktonic bacteria/microorganisms in natural environments predominate as adherent cells on different surfaces and form microcolonies or large populations. These multicellular and often multilayer communities are named biofilms and compared with citadels [16]. These communities were also compared with multicellular organisms, the component cells being embedded in a common matrix, differentiated and similar to eukaryotic tissues, with social behavior and properties based on the intercellular signaling by the QS mechanism [17,19,20]. The biofilm matrix surrounding bacteria makes them tolerant of stress conditions and resistant to all kinds of antimicrobials [17].

The coordinated action of the biofilm residents improves the ability of the community to attach to hosts and protects them from environmental stresses [21]. The multicellular nature of biofilms confers unique phenotypic abilities to the residing bacteria. Therefore, biofilms, not planktonic cells, are mainly responsible for the changes affecting their environment [10]. One example is the enormous impact of biofilms on human health. The U.S. Centers for Disease Control and Prevention (CDC) and National Institutes of Health (NIH) have estimated that bacterial biofilms are responsible for 65–80% of chronic infections, including burn wounds, chronic ulcers of limbs associated with diabetes, periodontitis, osteomyelitis, chronic wounds and cystic fibrosis lungs [22]. In addition, bacterial biofilms are able to evade the host immune system and are tolerant of usual clinical doses of antibiotics [23]. It has been demonstrated that biofilm-encased cells are 10 to 1000 times less susceptible to antibiotics than their planktonic counterparts [24] or such high doses are clinically and ecologically impracticable (being biohazard factors), which underlines the necessity of new alternative antibiofilm strategies [20,24].

### 2.1. Biofilm Formation and Dynamics

Even though biofilms were first observed and described by the father of microbiology, Antonie van Leeuwenhoek, a theory explaining biofilm formation and cyclic evolution was not stated until 1978 [15]. Direct observations (light microscopy and CLSM) have demonstrated that bacterial/microbial biofilms predominate in all nutrient-sufficient ecosystems, meaning all natural ecosystems, as well as in the food industry, water distribution systems and medical “ecosystems”, being responsible for these processes and creating problems of microbiological interest. It is demonstrated that biofilm-embedded bacteria are profoundly different from single, free, planktonic cells. Bacterial adherence and accumulation of signal molecules over a critical point activate or repress a large proportion of genes (40–60% of the prokaryotic genome has a different expression), so the biofilm cells change their behavior/phenotype to be very distinct from that of their free, planktonic counterparts. Each bacterial cell in a biofilm lives in a microniche which can be very different from the adjacent ones. It is appreciated that such a complex community has a primitive homeostasis, the sessile component cells living in a more stable, uniform environment, being more protected [19]. Some authors consider that these communities manifest a fundamental property of biological systems, named integrality [25]. A biofilm also has a primitive circulatory system (matrix channels allowing the supply of cells with water, oxygen/gases and nutrients) and metabolic cooperation, each cell living in a special surrounding environment [19].

The biofilm growth mode is predominant in all environments, being an alternative mode of growth to that represented by free or planktonic cells, these being only a transition phase and a form of dissemination [16,19,26]. The transition from single cells to a biofilm community is dependent on the synthesis of adhesins and other matrix components. A mature, multilayer biofilm is similar to a citadel or a tower with a 3-D structure. The matrix is crossed by small channels, which allow the transport of nutrients, signaling molecules and other metabolites, oxygen, water and waste, and with small spaces/cavities which provide shelter for other free microorganisms [16,17,20,27]. In the depth of biofilms, gradients of nutrients and oxygen vary from the top to the bottom of biofilms, and these gradients are the leading cause of decreased bacterial metabolic activity correlated with starvation and metabolic latency of cells (dormant or persister cells—the main cause of tolerance) and increased generation time of bacteria. Biofilm growth and exposure to stress conditions are also associated with an increased level of mutations (with survival value) as well as activation of QS-regulated mechanisms, such as virulence genes. So, the metabolic latency, bacterial cells in this state being insensitive to antibiotics, and conventional resistance mechanisms (chromosomal beta-lactamase, upregulated efflux pumps and mutations in genes coding for antibiotic target molecules) contribute to tolerance/survival/persistence of biofilm cells [24,27]. The mechanisms involved in this phenotypic resistance or tolerance, as well as those driving the transition from free-living bacteria to a differentiated biofilm, are still poorly understood. Nevertheless, dynamic and programmed metabolic responses allow the biofilms to react to local changes in nutrient levels, and metabolic adaptations contribute to phenotypic antibiotic resistance or tolerance of the community, suggesting novel therapeutic approaches to target biofilms [20,28].

Bacterial cell communication is realized by small, diffusible signal molecules or autoinducers (AIs), classified into two groups: AI 1 (acyl-homoserine-lactones (AHLs), specific for Gram-negative bacteria, peptidic AIs or AIPs for Gram-positive bacteria) and AI 2 (furanones), which allow a common bacterial language. The concentration of these AIs, dependent on cell density, is sensed by the community cells by the QS mechanism, involved in gene expression regulation, adaptation, biofilm formation and tolerance of biofilm-embedded cells to all types of antimicrobials [14,16,29].

At a cellular density over a critical point, the self-produced signals reach concentrations necessary for gene activation. This type of gene regulation was called quorum sensing (QS) and response (an adequate response at a certain cell density) [30].

A bacterial population (and other pathogens, or parasites) in an individual host has many of the characteristics of multicellular organisms. This status was proposed as a general bacterial feature. Intercellular communication and coordination of a microbial community are now known to be omnipresent among prokaryotes and contribute to their different phenotypes and social behavior [19,31]. Many different classes of signaling molecules have been identified in both Gram-positive and Gram-negative species. Bacteria have very complex signal transduction networks and are able to integrate these intercellular signals and give an adequate response by regulation of gene expression and cellular differentiation [32]. These populations or biofilms have a spatiotemporal multistep development, starting with a few sedimented cells, which adhere, multiply and colonize a surface and progress to microcolonies and then to a multilayer, mature biofilm. Finally, this mature biofilm, due to starved deep cells, starts to disintegrate and aggregates, or single cells are detached, floating again, and are able to disseminate and resume the process in another nutrient-rich, favorable environment [16,26,27].

It is proved that in these communities, the bacterial cells are not only well protected but also more efficient concerning their metabolic properties, both in terms of biosynthesis and biodegradation pathways [33]. In fact, this form of coexistence of bacteria in biofilms is considered the most successful and competitive expression of the prokaryotic genome [19]. The resistance/tolerance of biofilm-encased cells to antibiotics/antimicrobials is explained now by physical and physiological factors and genetic mechanisms, all together determining a behavioral or phenotypical change of a great part of the biofilm cells [26].

So, in all ecosystems, bacteria predominate as multicellular communities called biofilms, their cells being embedded in an extracellular matrix composed of polysaccharides and a proteinaceous part consisting of amyloid fibers. These fibers are stable and form aggregates similar to those specific to neurodegenerative diseases [20]. Such extracellular amyloid fibers, previously named *curli*, are produced, for example, by the uropathogenic pathotype of *Escherichia coli* (UPEC), as well as many other *Enterobacteriaceae*. It is proved that ring-fused 2-pyridones are able to inhibit curli biogenesis in UPEC strains and prevent the in vitro polymerization of the major protein CsgA of curli fibers [34].

However, bacterial amyloid proteins not only have a scaffold role but also have non-scaffold ones, contributing to biofilm formation and persistent biofilm-associated infections (BAIs), contributing to the formation of bridging cells during collective/aggregate migration, acting as toxins against bacterial competitors or even against host immune cells and playing an immunomodulatory role in their activities. All these effects can be inhibited by a new generation of antivirulence/antipathogenic drugs [20].

The biofilm cells are capable of various social or cooperative behaviors. At the same time, bacterial cells produce and respond to many signal molecules such as *quorum* sensors, toxins and siderophores and cooperate in biofilm formation by secreting the matrix components; in defense against host immune effectors and due to the proximity of cells, they exchange genetic information in a polyspecific or multiclonal population [35]. Due to this capacity to cooperate and share resources, the members of a microbial biofilm community were compared with social insects, because they are able to survive and transmit genes (horizontally or vertically, to the next generation) only as members of a community with similar characteristics [36].

As a model organism for Gram-positive bacteria and a member of the ESKAPE group, *Staphylococcus aureus* is a common pathogen responsible for not only soft tissue infections but also catheter-associated infections [37]. Its multiple adhesins belong to two groups: cell surface-associated molecules designated as Microbial Surface Components Recognizing Adhesive Matrix Molecules (MSCRAMMs)—represented by protein A, collagen-binding protein, elastin-binding protein and fibronectin—and soluble, secreted molecules called Secretable Expanded Repertoire Adhesive Molecules (SERAMs), represented by coagulase, fibrinogen-binding protein, extracellular adhesin protein (Eap) and extracellular matrix-binding protein (Emp) [38,39,40]. These adhesins and coagulase, which protect staphylococci against host immune effectors, all together transform a commensal, a member of the normal microbiota, into an opportunistic pathogen or a ”pathobiont”, able to produce skin and soft tissue infections, as well as invasive and persistent ones, mainly in immunocompromised hosts [40,41].

*P. aeruginosa*, as one of the six members of the ESKAPE group of bacterial pathogens, all of them being associated with high levels of AR, is an opportunistic pathogen and recognized as an etiological agent of chronic BAIs in immunocompromised patients, such as pulmonary infections of patients with cystic fibrosis or chronic obstructive lung disease and medical device-associated infections (urinary tract infections in patients with long-term catheterization, ventilator-associated pneumonia), and an important pathogen of polymicrobial wound infections. It is already demonstrated that antibiotherapy cannot eradicate these BAIs due to their behavioral resistance or tolerance and development through mutations of genetic resistance, favored by repeated exposure of bacterial strains to antibiotics [17,26].

The biofilm matrix formed by *Pseudomonas aeruginosa* strains contains mainly polysaccharides, proteins, extracellular DNA (eDNA) and lipids, the exact composition being dependent on strain, local conditions and biofilm’s age. The matrix is composed of polysaccharides such as alginate, *Pel* and *Psl*, as well as other components: type IV pili, fimbria, lectins, Fap amyloid fibers, eDNA and rhamnolipids involved in the formation of microcolonies and biofilms. All these matrix components contribute to the tolerance of *Pseudomonas aeruginosa* biofilm cells in response to the immune system [26].

Of great importance is the amplified resistance of biofilm cells, which are from 10 to 500 or even 1000 times more resistant/tolerant in response to antibacterial agents, concentrations totally impracticable, which requires the identification of effective alternative strategies [17,20,42,43,44].

### 2.2. Antibiofilm Strategies

At present, all available antibiotics are inefficient for the treatment of BAIs. It was demonstrated that biofilm-embedded cells are resistant/tolerant in response to usual clinical doses, calculated based on values of minimum inhibitory concentration (MIC) and minimum bactericidal concentration (MBC), determined by standard methods on free bacteria, in suspension. So, to reach an efficient value, the antibiotic dose has to be increased, but these higher doses are not always applicable due to their in vivo toxicity. Hence, it is critically important to design or screen antibiofilm molecules that can effectively minimize and eradicate BAIs. 

In the latest articles, researchers report various antibiofilm molecules discovered or tested to date, which include vegetal active compounds with antimicrobial and antibiofilm activities (isolated from herbs, leaves and tree bark, seeds and fruits) such as essential oils (EOs) and phenolic compounds, bacteriophages, bacteriocins/lantibiotics, antimicrobial peptides, nanoparticles (NPs), biological methods based on the interspecific antagonism (i.e., competition/probiotics or predatorism), physical modern methods based on light or ultrasound for biofilm removal and the use of new synthetic chemical compounds, as well as combined drug therapy (antibiotic with other antimicrobial agents, i.e., nanoparticles, EOs or QS inhibitors (QSIs)), enzymes, chelating agents and immunological methods.

The use of such new compounds is now based on their complete characterization, regarding their structures, mechanisms of action, MICs, MBCs, minimum biofilm inhibitory concentrations (MBICs) and cytotoxicity.

Many bacteria produce several adhesins with different receptor specificities. Many studies have shown that even the inhibition of a single adhesin can sometimes be sufficient for transforming a pathogenic strain into a non-virulent one [45], and such a substance/drug is called antipathogenic. In this context, the research currently focuses on the discovery of new antibiotics, as well as innovative drugs/therapies and alternative strategies for the efficient combat of MDR pathogens and biofilm formers.

Table 1 summarizes these new antibiofilm strategies (accompanied by a selection of references, mainly original papers) that will be briefly characterized in the second part of this review.

## 3. Resistance, Virulence and Bacterial Fitness

The expression of resistance and virulence features is associated with biological costs for bacteria carrying these genes, and it is variable, depending on the species involved, the virulence and resistance mechanisms and their diversity, and the habitat, be it outside or inside the host organism. Even though the AR genes are not properly virulence genes, they represent a survival advantage for pathogens and contribute to an amplified virulence and fitness of MDR strains, nowadays representing a huge challenge for the medical field [14,112].

The existence of these high-risk clones necessitates the development of new strategies, namely new antibiotics or other new types of drugs, as well as novel and rapid diagnostic methods able to detect resistance and virulence markers. Such novel therapeutic ways and diagnostic tests could contribute to solving the increasing problem of these superbugs, which benefit from an extra advantage, to the detriment of the host [113].

The pathogenic potential of bacterial pathogens is dependent on one or more virulence factors, determining the ability of a bacterial strain to enter, localize, colonize and resist host defense mechanisms, generating a disease manifested by different symptoms. The clinical picture of an infectious disease depends on the major properties of a pathogen (pathogenicity and virulence) and on the host status at the time of their meeting (host age, immune status, diet, environmental conditions), which has a decisive role in host susceptibility and disease evolution. The virulence factors used by the pathogenic microorganisms include different aggressins (invasins, toxins, secretion systems), adhesins (EPS components, pili, fimbriae, curli or amyloid fibers), biofilm formation capacity and siderophores [114,115].

Some virulence genes are located on chromosomes, i.e., some adhesins, but others are located in plasmids or other mobile genetic elements. So, virulence and most AR genes are often co-located and can be transmitted horizontally [10].

The phenotypic and genotypic characterization of AR and of the virulence genes in MDR Gram-negative bacilli are of great importance for reducing the epidemiological challenge in the management of healthcare-associated infections [116]. It is well known that there are no new antibiotics against Gram-negative bacteria; their specific cell wall structure, with an outer membrane (OM), the OM proteins and their modified permeability, and efflux pumps all define their intrinsic resistance [117]. However, the efflux pump activity and the OM proteins are associated with several virulence factors, according to many large protein databases (UniProt; VFDB) [118].

For example, one of the most clinically relevant pathogens is *Pseudomonas aeruginosa*, which is responsible for the high incidence of MDR infections in immunodeficient patients. The major concern regarding this opportunistic pathogen is its great adaptability, correlated with many virulence factors and AR mechanisms. So, there is an increased need for developing new strategies for preventing and managing infection with MDR strains. The biofilm formation and QS mechanism are intensively investigated, together with the type III secretion system (T3SS) and the secreted toxins [119]. Recent studies concerning the biofilm-forming bacteria suggested the associative effect over the mechanisms of AR of both inflammatory endotoxins/LPS (with intense action in a biofilm formed on tissues) and the expression of the stress genes [120,121]. According to some authors, the QS mechanism and signal molecules are associated with biofilm formation and intrinsic resistance mechanisms in *P. aeruginosa*, but even environmental factors play a major role in the activation of virulence genes [122].

Among the same group of non-fermentative Gram-negative bacilli, *Acinetobacter baumannii* is also studied regarding its biofilm formation capacity and QS mechanisms related to virulence gene expression and AR. A current preoccupation is to identify the best drugs that can target such mechanisms, aiming to reduce the excessive antibiotic burden that could possibly increase the AR [123]. A series of studies on *A. baumannii* strains have emphasized the presence of multiple resistance genes that now cover almost all classes of antibiotics. The research focus is also on other virulence factors, such as OmpA, often correlated with β-lactam resistance [124].

Another representative pathogen from the group of Gram-negative bacilli is *Klebsiella pneumoniae*; its pathogenic potential is influenced by the expression of its virulence factors: pili (especially coded by *mrk* and *fim* genes), efflux pumps, LPS, T6SS, capsule, siderophores and other proteins. Recently, it was shown that the ability of *K. pneumoniae* to colonize the host is favored by the co-occurrence in some resistant strains of a blaKPC-carrying plasmid and pLVPK-like virulence plasmid, and even by hybrid resistance- and virulence-encoding plasmids in hypervirulent, superbug strains of *K. pneumoniae* isolated from patients with bacteriemia [125].

Askoura and Hegazy (2020) proved that intracellular survival of ciprofloxacin-sensitive and -resistant *Salmonella* was markedly reduced upon treatment with a sub-MIC of ciprofloxacin (CIP). The results were confirmed using immunostaining indicating an inhibitory effect of a sub-MIC of CIP on *Salmonella* intracellular survival. By RT-qPCR, it was proved that the expression of genes coding for the *Salmonella* type 3 secretion system (T3SS) decreased after bacterial exposure to a sub-MIC of CIP. Moreover, bacterial exposure to a sub-MIC of CIP drastically reduced the expression of both sifA and sifB, coding for *Salmonella* filament formation inside the host. In an infection model, in mice inoculated with *Salmonella* exposed to a sub-MIC of CIP, the bacterial killing capacity was reduced in comparison with mice infected with untreated bacteria. These results indicate that, in addition to its bactericidal effect, a sub-MIC of CIP could inhibit *Salmonella* intracellular survival, virulence gene expression and pathogenesis. These results are very significant because the species of *Salmonella* are facultatively intracellular, invasive pathogens, and many species of *Salmonella* are common enteric pathogens worldwide [126,127].

The virulence of pathogens is also influenced by their capacity to actively move toward favorable niches. For instance, some *P. aeruginosa* mutants, designated surface attachment defective (sad) have been described, some of them being defective for flagellar motility and others for twitching movement, motility being necessary for biofilm development. The strains defective in flagellar motility appeared to be inhibited in the initial interactions with a surface. The second group of strains was defective in the biogenesis of type IV pili, known to be implicated in surface-associated movement or twitching motility. Strains unable to synthesize functional type IV pili are able to attach to the surface and form a monolayer similarly to the wild type, but they are not able to form microcolonies, a characteristic of early biofilm formation by *P. aeruginosa*. So, twitching motility is necessary for the assembly of microcolonies and substrate colonization [128].

It was proved that mobility and chemotaxis are key factors that also mediate host invasion in *Salmonella* serovars. Generally, for enteric pathogens, such as EPEC pathotype cells, flagellar motility is very important for adherence to the host intestinal epithelial cells, favoring the movement across the mucus layer and the contact with host cell receptors [129].

*Helicobacter pylori* also requires motility to cross the mucus layer toward the epithelial cells of the stomach. Due to the increasing resistance of this pathogen to antibiotics, there is a growing need for new strategies for its effective eradication. It is already known that the inhibition of the QS system in most microbial pathogens leads to decreased virulence. However, a different reaction is observed in *H. pylori* when interfering with the production of AI-2 which initiates biofilm formation and increases bacterial survival. It is considered that there is an alternative way to control the physiological changes of *H. pylori* exposed to environmental stressors. Other compounds probably involved in the expression of *H. pylori* virulence factors, such as diffusible signal factors (DSFs) represented by fatty acid signal molecules, were identified. DSFs enhance *H. pylori* transition into a non-motile state, correlated with bacterial transformation into a more resistant coccoid form, able to initiate biofilm formation, accompanied by bacterial cell protection against adverse environmental factors (low pH, oxidative stress, local immune system) and limiting the diffusion of an effective concentration of antibiotics [130].

It is accepted that the adaptive and survival mechanisms of *H. pylori* in the stomach are also linked to its ability to adhere and form biofilms (outside and inside the host). In the last two decades, the attention of researchers has been focused on this aspect of biofilm formation on the stomach mucosa and the consequent therapeutic difficulties of *H. pylori* infection related to virulence and adaptive responses, such as morphological transformation, membrane vesicle secretion, matrix secretion, efflux pumps and intercellular communication. In a study performed by Krzyżek and coworkers (2022), in a flow system of cultivation, it was observed that strong biofilm formers also produced significantly more eDNA and in particular a rich protein matrix of biofilm in comparison with the weak biofilm producers. Moreover, it was observed that strong biofilm producers manifested a higher tendency for autoaggregation and morphostructural differences (a greater cellular aggregation, shorter or coccoid cells, and a higher amount of OMVs and flagella too) in comparison with weak biofilm formers. The authors concluded that resistance to clarithromycin in clinical strains was associated with many phenotypical features all favoring the ability to form strong biofilms [131,132].

The evolution of the pathogen colonization steps and the infectious process are modulated by natural selection and the capacity of a pathogen to adapt to the host environment, making a balanced allocation of energy resources necessary for growth, colonization, survival and multiplication, all these processes leading to a maximized reproductive fitness of the pathogen, with an influence on the infection’s evolution [36].

## 4. Drugs as Antibiotic Alternatives or Complements

The environmental microbiota is the primary source of current natural antibiotics (mainly produced by various species of the actinobacteria group, most of them belonging to *Streptomyces* spp.). Generally, natural antibiotics are part of interspecies antagonism, meaning that they are primarily produced in very abundant, dense communities, such as soil or seawater micropopulations, as well as human microbiota (Table 2).

### 4.1. Antimicrobial Peptides and Enzymes

Antimicrobial peptides (APs) or host defense peptides seem to be viable alternatives to traditional antibiotics because they present several advantages over others [154]. In addition to antimicrobial activity, these compounds show immunostimulatory and anti-inflammatory properties [155], which can provide a more robust and appropriate treatment of bacterial infections. The ability of APs to neutralize some endotoxins (in in vivo models) is another of their assets [156], to which is added their property to prevent the formation of bacterial biofilms [157]. Furthermore, recent studies have shown that they can potentiate the effects of other antibiotics, and it is feasible to develop medical products with APs for topical application, also considering the antioxidant potential of some of them [158,159]. Currently, attempts are being made to overcome some obstacles, such as bacterial strategies for resistance [160] and their toxicity through various strategies such as their chemical modification (especially of terminal groups), the use of nanotechnologies and bioinformatics studies [157], and some APs are already in clinical trials [157].

A promising strategy is based on matrix-degrading enzymes, able to dissolve the matrix and biofilm architecture and integrity (e.g., dispersines, DNase, alginate lyase), because they are able to recover the cells’ susceptibility [27,161] or to degrade the signal molecules’ quorum-quenching (QQ) enzymes and inhibit the expression of genes controlled by cell density, such as virulence genes [123,162]. Examples of QQ enzymes are lactonases, decarboxylase, acylase and deaminase, and all are able to inactivate AHLs [133,163].

As it was previously mentioned, an important bacterial group of antimicrobial producers is represented by different species of *Bacillus*, which produce a wide range of antimicrobial substances, including peptide and lipopeptide antibiotics and bacteriocins too [164] (see also Section 4.2).

Another example of an enzyme with antibacterial properties is the one produced by *Serratia marcescens*, a protease named serrapeptase (SPT). Its antibacterial properties are similar to or even superior to those of many antibiotics. For example, Katsipis and Pantazaki (2023) reported that SPT has antibiofilm activity, demonstrated against two reference strains of *S. aureus*—a methicillin-susceptible strain, MSSA (ATCC 25923), and a methicillin-resistant strain, MRSA (ST80). The authors explain the antibiofilm activity of this enzyme by the peptidic nature of the cell wall and extra-wall components of *S. aureus* such as peptidoglycans (PGs) and amyloid proteins, which are essential for biofilm development (together with the lipoteichoic acids) and for biofilm tolerance of *S. aureus* strains, including methicillin-resistant *S. aureus* (MRSA) ones. So, SPT impairs the biofilm development of staphylococci. It is proved that SPT treatment reduces biofilm formation and bacterial viability, affecting the alkaline-phosphatase activity and phospho-homeostasis [161].

Since 2010, the use of enzymes able to disintegrate the biofilm matrix (e.g., DNase and alginate lyase) and the use of QSIs have been considered promising strategies for BAIs, and both are able to increase the susceptibility of biofilm cells to antibiotics [27].

Recently, a study reported about the biofilm produced by *Pseudomonas aeruginosa*, its matrix acting as a protective diffusion barrier against antibiotics and immune system effectors [26]. The most abundant extracellular matrix polysaccharide formed by *P. aeruginosa* is alginate, a homo- or hetero-polysaccharide composed of β-D-mannuronate and α-L-guluronate monomers. The degrading enzymes of alginate, named alginate lyases, have been proposed as potential therapeutic agents, able to disintegrate *P. aeruginosa* biofilms. However, it should be mentioned that there are contradictory scientific reports concerning the efficacy of these enzymes against biofilms or of combinations between these enzymes and antibiotics. The most favorable results in dissolving biofilms were obtained with commercial crude extracts containing alginate lyases produced by bacteria belonging to *Flavobacterium multivorum* and *Sphingomonas* spp. [165].

Other antibiofilm and antivirulence drug targets are some enzymes such as bacterial sortases (Srts), small molecules essential for the secretion and anchoring of many wall or extra-wall proteins/adhesins. The inhibitors of Srt belong to vinyl sulfones, 3-aryl acrylic acids and derivatives, flavonoids, naphthoquinones, anthraquinones, indoles, pyrrolomycins, isoquinoline derivatives, aryl β-aminoethyl ketones, pyrazolethiones, pyridazine, benzisothiazolinones, etc. [166]. This strategy is considered one of the most promising antivirulence ones against Gram-positive bacteria. Srts were identified in almost 600 bacterial species [167], and in their absence, the adhesion to the substrate and infectivity were proven to be drastically reduced [168]. Other inhibitors of sortases (Srts) are the peptidomimetic molecules (diarylacrylonitriles) which exert a strong inhibitory effect against *S. aureus*, inhibiting the secretion and anchoring of many wall proteins and adhesins, including pili. Due to their high level of conservation in Gram-positive bacteria, sortases are considered good targets for antipathogenic drugs [169].

Targeting bacterial enzymes is not a new approach, but identifying compounds that target metalloenzymes, especially carbonic anhydrase inhibitors (CAIs), has been intensively studied in the last ten years. For example, the bacteriostatic effect of CAIs was demonstrated for *Helicobacter pylori*, *Escherichia coli* and *Mycobacterium tuberculosis* [170]. In addition, CAI sulfonamides such as acetazolamide [171], its derivatives [172] and dorzolamide [173] have recently been shown to be effective against vancomycin-resistant *Enterococcus* (VRE). Moreover, it is considered that these CAIs have little potential to develop resistance, and, in some cases, they also have the advantage of clinical use for over 70 years (for other pathologies).

Other enzymes that are targeted in recent studies, identified in mammals and among the candidate targets for which other inhibitory/blocking molecules that could be used clinically have not yet been discovered would be the following: (i) prokaryotic bifunctional FAD synthetases (FADSs) responsible for the synthesis of flavin mononucleotide (FMN) and flavin adenine dinucleotide (FAD) [174]; (ii) bacterial transglycosylase (TG) [175] and disulfide oxidoreductase (DsbA); (iii) redox enzymes represented by the DsbA/DsbB system for example that catalyze the formation of disulfide bonds for many virulence factors, essential for most pathogens [176].

### 4.2. Bacteriocins

Bacteriocins have been known for a long time (almost a hundred years—Gratia, 1925), and it is estimated that 99% of all prokaryotes (bacteria and archaea) are able to produce one or more bacteriocins. The ability of bacteriocins to inhibit the activity of certain bacteria and to be noted—without distinguishing between antibiotic-resistant and -sensitive strains—is of great interest, especially since the phenomenon of antibiotic resistance has reached a pandemic level, with bacterial strains that have developed multidrug and even pandrug resistance (PDR) to most common antibiotics [177].

**Figure 1:**
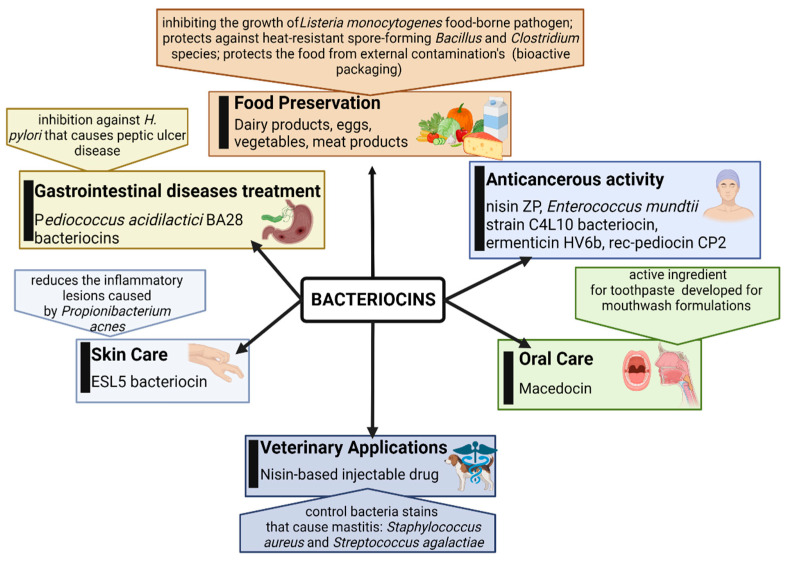
Commercial bacteriocins and their applications (original, BioRender design).

Only a few characterized bacteriocins have been introduced into commercial products (Figure 1) [178], most of them, especially the bacteriocins produced by lactic acid bacteria (LAB) being extensively and safely used in food fermentation [179]. Nisin (a bacteriocin produced by *Lactobacillus lactis*, subsp. *lactis*) is a good example, being approved as a food additive since 1960 in more than 50 countries for its antibacterial effect against Gram-positive, sporogenic pathogens belonging to *Clostridium* spp. and *Bacillus* spp. [180]. Thereafter, other new bacteriocin producers were searched for, and in the late 1990s, some GRAS *Bacillus* spp. were discovered, their bacteriocins having a broader antimicrobial spectrum than those produced by LAB [181].

*Bacillus* spp. produce a wide range of antimicrobial substances, including peptide and lipopeptide antibiotics, and bacteriocins, classified into three main classes (with subclasses): class I includes lantibiotics, or post-translationally modified peptides which are the best-characterized bacteriocins; class II includes non-modified peptides; class III includes bacteriocins with m.w. greater than 10 kDa [133,164].

According to their different chemical structures, bacteriocins also have various mechanisms of action, such as pore formation, cell disintegration or protoplasm vesicularization with bactericidal effect [182]. Bacteriocins synthesized by *Bacillus* spp. are becoming more important than most LAB bacteriocins because they often manifest a broader spectrum of activity, inhibiting not only Gram-positive bacterial species, but also Gram-negative bacterial species and yeasts/microfungi, including different pathogenic species [164]. For example, helveticin-M is a class III bacteriocin produced by *Lactobacillus crispatus*, and it possesses antimicrobial activity against *Staphylococcus aureus*, *S. saprophyticus* and *Enterobacter cloacae*, having as a main destructive target the cell wall of Gram-positive bacteria and the outer membrane of Gram-negative bacteria [51].

*Bacillus* spp. also produce other antimicrobial substances; those which are not yet well characterized are termed bacteriocin-like inhibitory substances, especially when the peptidic composition is not confirmed [133,164].

### 4.3. Bacteriophages

Bacteriophages are present in all ecosystems where their specific hosts, bacteria, are present. Despite human microbiota biodiversity, especially intestinal microbiota biodiversity (bacteria, archaea, microfungi/yeast, protozoa), and its cellular density estimated at 10^14^, the virome diversity and abundance are even more significant, the number of viruses being estimated at 10^15^, most of them being bacteriophages, with an important contribution to the microbiota eubiosis status [183]. There are reports indicating that enteric viruses have functional and genetic relationships with the host and other components of the intestinal microbiome. The viral community is in turn regulated by normal microbiota by processes termed transkingdom interactions, which is a new paradigm in intestinal immunity to a viral infection which could lead to some therapeutical applications [184].

Phage therapy for bacterial infections was glimpsed and even experienced by the co-discoverer of bacteriophages—Félix d’Hérelle (1917) [185]. The actual premises of the phage therapy development are the large number of different bacterial strains that are already successfully targeted. Phage therapy to treat bacterial infections is not a new treatment strategy but gained more attention in direct relation to the AR phenomenon. Bacteriophages exhibit a significant bactericidal effect, especially the lytic bacteriophages by their capacity to invade the bacterial cell and kill it [186]. Temperate phages could also be used as genetic tools to increase bacterial susceptibility to antibiotics based on their transduction mechanisms, but certain gene types such as drug resistance and bacterial virulence factor genes are easily spread through this process [186]. 

Monophage products have been used in the last few years, but researchers expect to accomplish multistrain or even multispecies products. In order to increase the phage “depth” of activity, they have already prepared phage cocktails more efficiently and avoided the potential of bacteria to evolve phage resistance. Such a cocktail must include at least two phages, both able to infect a single strain, and against which bacterial mutation is relatively rare [187].

The potential for a monophage to infect and control a specific bacterial pathogen is a function called “breadth of that phage’s host range” which is pharmacologically similar to the spectrum of activity of a chemical antibacterial agent [188]. One general means of increasing the spectrum of activity breadth and efficiency of a monophage is to use combinations of therapeutical solutions. For instance, phages can be combined with other types of agents, such as antibiotics, or with other phages specific for other host bacteria; in this last case, the combination is now named a polyphage or phage cocktail [187,189,190].

A benefit of phage therapy is its high selectivity, which reduces the side effects, mainly the dysbiotic effect on normal microbiota. At the same time, this characteristic can be limitative, and a specific phage can become inefficient on similar isolates of the same pathogen. This inconvenience can be bypassed by phage cocktails screened against a large number of isolates or by the engineering of phages to increase their spectrum of activity. The resistance to a phage by a bacterium is considered to be a consequence of their long-time co-evolution [191].

The phage cocktail spectrum of activity may be extended in order to target different bacteria types or inhibit their potential to develop phage resistance. Some authors make a distinction between the functions of a cocktail’s breadth and a cocktail’s depth of activity [187]. In further detail, the depth of activity resides in the potential of a single bacterial mutation to lead toward the resistance of bacterial cells to phage lysis of two or more different phage types; this property is named cross-resistance [192].

Phage cocktails can be composed of individual phages which target multiple species and genera too, and these products can be used empirically to treat polymicrobial diseases, especially skin/soft tissue infections. In this case, a product named Pyophage has been developed to treat infections produced by strains belonging to *Enterococcus* sp., *Escherichia* sp., *Proteus* sp., *Pseudomonas* sp., *Staphylococcus* sp. and *Streptococcus* sp. [187].

As infections with *Acinetobacter baumannii* raise serious problems, many clinical strains being MDR, using phage therapy is sometimes the only option. Recently, a group of researchers from Poland searched for phages specific to MDR *A. baumannii* strains in different waters (environmental, municipal and hospital wastewater samples). They succeeded in isolating 12 phages specific for *A. baumannii*, but only 1 was a lytic phage, and it showed the capacity to form relatively large plaques with a clearly marked “halo” effect in vitro [193].

Bacteriophages, mainly their purified enzymes—bacteriolysins or simply lysins—could be used especially in recombinant form as antibacterial agents [194]. These enzymes can be used extracellularly and combined with antibiotics as enzybiotics, which can also attack the metabolically latent or persister cells, being a potential antibiofilm strategy. These phage-derived enzymes have proved their efficiency in experimental in vivo infections caused by Gram-positive and Gram-negative bacteria, and they have already entered clinical trials [195].

The bactericidal effect of lysins on Gram-negative and Gram-positive bacteria has recently been demonstrated for P9LY, an enzyme produced by *Shigella dysenteriae* infected with the PSD9 phage, as well as for the lysins produced by *Meiothermus bacteriophage* (from thermophilic media) [196,197]. Moreover, according to São-José and coworkers (2022), the lytic enzymes of effective phages on Gram-positive bacteria, anti-streptococcal endolysins, proved an in vivo bactericidal effect [198].

### 4.4. Competition—Probiotics

In the Joint FAO/WHO Working Group on Drafting Guidelines for the Evaluation of Probiotics in Food (2002) [199], a new definition of a probiotic was adopted, stating that “a probiotic = a viable microbial food component which has a demonstrated benefit on human health when given in specific amounts” [200]. So, probiotics, as mono- or multispecific products, are essential dietary viable components that can reduce the risk of infectious diseases due to their main direct and indirect mechanisms: production of antimicrobial substances (such as organic acids, H_2_O_2_, bacteriocin-like substances, bacteriocins, biosurfactants) [201], competition for nutrients, adherence to host receptors, competitive rejection of pathogenic microbiota, and stimulation of nonspecific and specific host immune responses [202]. Apart from direct implications in promoting intestinal homeostasis by maintaining the symbiotic balance of the gut microbiome and the host, probiotics have proven many other beneficial effects and protective mechanisms against infectious diseases. For instance, in a rat gastrectomy model, high-dose probiotics (*Lactiplantibacillus plantarum*, *L. rhamnosus* and *Lacticaseibacillus acidophilus*) produced a downregulation of inflammatory protein levels caused by the activation of Toll-like receptor 4 (TLR4)/nuclear factor kappa B (NF-κB) signal pathway [203]. Moreover, probiotics produce short-chain fatty acids (SCFAs) by dietary fiber fermentation, altering the acid–base environment in the intestinal lumen and thereby inhibiting the growth of pathogens [201].

The discovery of communication systems regulating bacterial virulence has afforded a novel opportunity to control infectious bacteria without interfering with growth. For example, Chifiriuc and coworkers [204] have studied the effect of phenyllactic acid (PLA) (a major LAB metabolite) in sub-MICs on the pathogenicity of *Pseudomonas aeruginosa* in mice. The study showed that the animals infected with PLA-pretreated cultures exhibited good survival rates (0% mortality) and almost complete abolition of the pathogen’s invasive capacity. Thus, it was demonstrated that *D*-3-phenyllactic acid (PLA) can act as a potent antipathogenic drug, without interfering with bacterial growth, also having the capacity to eliminate the pathogen from the animal host [204].

It has also been demonstrated that probiotics may stimulate innate immune pathways such as those stimulated by respiratory viruses, thereby modulating the antiviral immune response [205]. In this context, there are some studies concerning the role of probiotics in restoring the oropharyngeal microbiota as a complementary method against SARS-CoV-2. Characterization of the oropharyngeal microbiome in recovered COVID-19 patients showed an increased abundance of the butyrate-producing *Fusobacterium* that could promote intestinal mucosal barrier repair, which can contribute to recovery from COVID-19, and a decreased abundance of the lipopolysaccharide (LPS)-producing opportunistic pathogen *Leptotrichia*, a risk factor for an induced systemic proinflammatory status during COVID-19 development [206].

**Figure 2:**
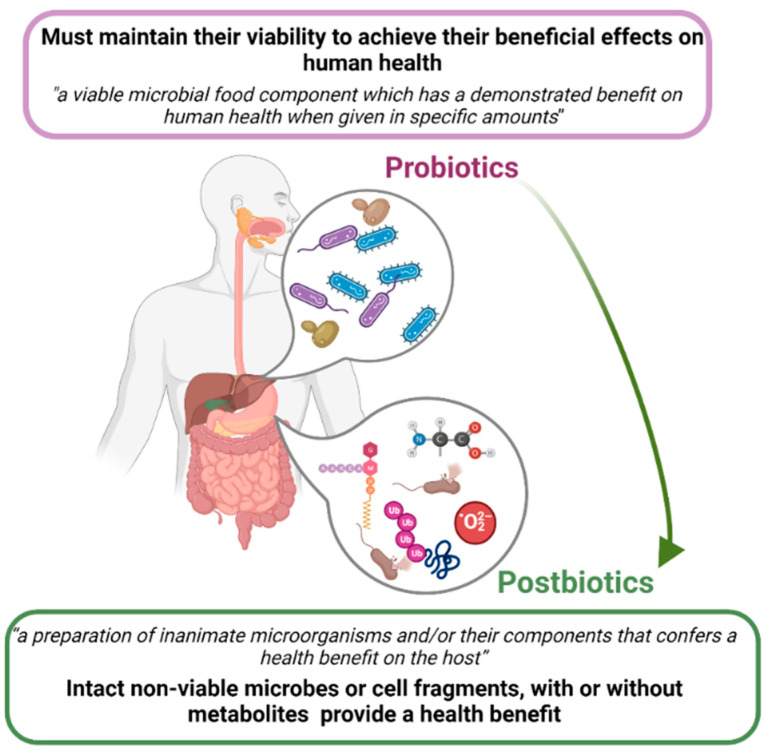
Diagram illustrating the concepts of probiotics and postbiotics (original, BioRender design).

To achieve their beneficial effects on human health, the probiotic strains must maintain their viability, which can be affected by inappropriate conditions of the processing, storage, distribution, preparation and administration [207]. Since probiotics raise a series of problems related to the safety of certain risk groups such as children or immunocompromised people, a new concept of postbiotics is defined and proposed as an alternative for clinical applications (Figure 2).

The new concept of postbiotics was defined in 2021, bringing together a series of terms already used in the literature, such as paraprobiotics, ghostbiotics, heat-inactivated probiotics, non-viable probiotics, cell fragments and cell lysates, all terms referring to microbial components incapable of colonizing the host, to spread virulence and/or antibiotic resistance genes from live cells to the gut microbiota via horizontal transfer [208]. Thus, in 2021, postbiotics were defined as a “preparation of inanimate microorganisms and/or their components that confers a health benefit on the host”, according to the International Scientific Association of Probiotics and Prebiotics [209].

In a comparative study regarding the beneficial effects of probiotics and postbiotics in mitigating dextran sulfate sodium (DSS)-induced colitis using a mouse model, Zhang et al. (2022) demonstrated that a postbiotic product (represented by non-viable *Bifidobacterium adolescentis* B8589 powder) exhibited a stronger ability to modulate the gut microbiota and its functional metagenomic potential compared with a probiotic product (represented by live *Bifidobacterium adolescentis* B8589) [210]. Short-term administration of *Lactobacillus gasseri* CP2305 improves stress-associated symptoms and clinical symptoms in healthy young adults and in patients with irritable bowel syndrome [211].

A randomized controlled trial was performed by Satomi et al. (2021) in order to investigate whether a combination of heat-killed *Levilactobacillus brevis* KB290 (KB290) and β-Carotene (KB290 + βC) could reduce the incidence of influenza and common colds as well as alleviate clinical symptoms in healthy Japanese adults. They demonstrated, in a first large-scale human clinical trial, that these combined food components might be a possible candidate for protection against seasonal influenza virus infections in humans aged <40 years [212].

### 4.5. Predatorism

The exploitation of antagonistic relationships, including the predation of pathogenic bacteria, is also considered a solution for the global problem of AR. A well-known predatory bacterium, discovered 60 years ago [213] when isolated from a soil sample during an experiment attempting to isolate bacteriophages, is the species named *Bdellovibrio bacteriovorus (gr. Bdellos* = leech, parasite) which presents a life cycle similar to the phage cycle. *B. bacteriovorus* is a predatory species, cultivable in the lab, Gram-negative, mobile, and present in aquatic and terrestrial environments, acting as a community balancer [99,214]. The cells of this predator live as free bacteria and are able to attack other Gram-negative bacteria, penetrating into periplasmic space (this form is named bdelloplast).

There are reports about the therapeutical value of predatory species, for instance, for treating chronic bacterial infections, associated with biofilm formation, such as periodontal infections [215,216].

*Bdellovibrio bacteriovorus* is also present in animal and human guts. Moreover, it is present in abundant populations only in healthy persons and in a reduced number in patients (e.g., IBD, Celiac disease). Another observation is its presence as mucosa-associated cells, with a major dominance in the duodenum and a decreased dominance along the gut. *B. bacteriovorus* is considered by some authors as a new therapeutic strategy, having the potential of a probiotic able to balance the intestinal ecosystem [99].

Current studies suggest that *B. bacteriovorus* can be used as an alternative solution to antibiotherapy, being a real “living antibiotic”. Researchers are now interested in its interaction with biofilms and MDR strains, and supplementary studies about the interaction with host immune response, susceptibility of different pathogens to this predator, and in vivo models are necessary [214]. There are already data about the remarkable potential of *B. bacteriovorus* to kill MDR bacteria, such as the group of the most life-threatening pathogens, often implicated in the etiology of nosocomial infections, such as the *Enterobacter* genus, *Klebsiella pneumoniae*, *Staphylococcus aureus*, *Acinetobacter baumannii*, *Pseudomonas aeruginosa* and *Escherichia coli* [214,217].

### 4.6. Plant Extracts with Antimicrobial, Antibiofilm and Antipathogenic Effects

Among the proposed solutions for MDR pathogens, a strategy with chances of success is represented by the screening of therapeutics able to modulate key phenotypes of microbial virulence and control the progression of the infectious process without selecting resistant mutants. In this category of new therapeutics, natural compounds are ideal candidates, especially because their therapeutic activity is already proven. The medicinal properties of plants have been known since ancient times, but now is the moment to pass from ethnomedicine to the scientific phase. So, medicinal plants are now investigated at the molecular level, in order to identify the mechanisms of action, efficiency and lack of cytotoxicity, since their use has to be scientifically based, in definite amounts, and for a specific target, completely characterized, using the same methods as in the case of allopathic drugs. The potential synergistic activity with antibiotics should also be explored [218,219]. However, not only medicinal plants have such properties, but also other plants (edible plants, spices) were investigated and proven to contain antimicrobials. Thus, plants are considered an important source of bioactive substances; all plants have immune defense mechanisms mediated by anti-infectious phytocompounds, such as phytoanticipins and phytoalexins, and the more recently described QSIs. The QSIs, even when used in subinhibitory concentrations (sub-MICs), exhibit an indirect antimicrobial effect manifested by inhibiting bacterial cell-to-cell communication by the QS mechanism and coordinated expression of virulence genes depending on cellular density. The use of QSIs could represent an efficient and intelligent strategy to control resistance/tolerance, virulence, and colonization/biofilm formation, without selective pressure and other side effects [13,220,221,222,223,224].

For instance, among edible plants containing QSIs and having antibiofilm potential is *Caparis spinosa*; its extract inhibited the biosurfactant production in *Pseudomonas aeruginosa* PAO1, swimming and swarming motility, EPS synthesis and biofilm formation in *Escherichia coli*, *Proteus mirabilis*, *Serratia marcescens* and PAO1, and all these effects were manifested without affecting the bacterial growth [225].

Another extract from a common herb/spice (*Anethum graveolens*) was tested on *S. marcescens*, an opportunistic pathogen frequently associated with urinary tract infections (UTIs) and often multidrug resistance, and it exhibited antibiofilm formation activity and antipathogenic potential, interfering with the QS system and reducing virulence factor production (the downregulation of virulence genes being assayed by molecular tests). One of its major active principles, 3-O-methyl ellagic acid, is able to inhibit the prodigiosin and protease synthesis in *S. marcescens* [226].

In addition, plants used as spices and flavors, such as vanilla extract (*Vanilla planifolia*), demonstrated the potential of QS inhibition on the Tn-5 mutant of *Chromobacterium violaceum CV026*, inhibiting violacein production in a concentration-dependent manner. Vanilla-containing food might promote human health by inhibiting QS and preventing bacterial pathogenesis [227]. Sotolon is an aromatic compound that gives fenugreek its typical smell. Aldawsari and coworkers (2021) conducted in vitro and in vivo experiments proving the antipathogenic activities of sotolon (in sub-MIC) against *Pseudomonas aeruginosa* [228]. However, using natural products has some limitations due to their low availability and stability, high volatility and great diffusion ability that do not support their implementation in the current medical practice. These features make it necessary to develop vectorization and delivery agents to improve their efficiency and optimized assay methods adapted to their specific properties. However, the research efforts are fully justified by their great potential [4].

Some extensively studied options with multiple applications are essential oils and polyphenols.

#### 4.6.1. Essential Oils (EOs)

The essential oils (and their components) obtained by the hydrodistillation of aromatic plants have been shown to have antimicrobial properties. Previous studies have shown that certain EOs or purified EO-derived compounds interfere with microbial virulence, reducing biofilm formation and modulating the expression of virulence genes in bacteria [229].

Unfortunately, some of their properties, such as volatility, instability and high doses required for an efficient therapy, currently limit their use in biomedical practice. Still, these drawbacks could be overcome by combination with nanocarriers or other controlled delivery systems of bioactive substances at the target sites. So, many studies are focusing on the research of the antimicrobial activity of EOs and their components alone against biofilms [230,231,232] or combined with nanocarriers/nanomaterials [233,234].

EOs are mixtures of molecules such as terpenoids, phenol-derived aromatic components and aliphatic components. Their quality, quantity and composition can vary depending on plant species, subsp., growth conditions (seasonal variation, climate, phenophase at the moment of harvesting), plant organ, age and oil extraction method [235].

For example, an EO mixture of *Artemisia annua* exhibited antimicrobial properties against reference strains of Gram-positive and Gram-negative bacteria and yeast strains; EOs used in subinhibitory concentrations were proven to inhibit the phenotypic expression of different virulence factors—soluble (hemolysins, gelatinase, DNase, lipase and lecithinase) and cell-associated ones/adhesins—and adherence capacity [219].

Therefore, the antimicrobial activity of EO mixtures and other fractions may be different, so researchers focus on their main components, such as carvacrol, thymol and eugenol, with proven antibacterial properties [236].

For instance, carvacrol (5-isopropyl-2-methyl phenol) is a natural compound, a phenolic monoterpenoid found in leaves of some plants including oregano, thyme and pepperwort. Many studies describe carvacrol as a compound with a wide range of bioactivities, including antimicrobial, antioxidant and anticancer activities. As an antimicrobial compound, it showed activity against Gram-positive and Gram-negative bacteria and fungi, as well as against planktonic and sessile human pathogens [237,238]. The activity against sessile bacteria indicates that carvacrol interferes with the QS mechanism, which had already been demonstrated by Burt and coworkers (2014), working on the reference strain *C. violaceum* ATCC 12472 exposed to a sub-MIC of carvacrol that reduced expression of the gene *cviI* (coding for the N-AHL synthase), synthesis of the pigment violacein, chitinase activity (both regulated by QS), and biofilm formation; these results clearly indicated that these effects of carvacrol are correlated to the interruption of the QS mechanism [239].

Carvacrol and its isomer thymol (isopropylmetacresol, 2-isopropil-5- methylphenol) are recognized for their antibacterial and antifungal activities [240]. Thymol is also a constituent of thyme essential oil (up to 50%), found in plants such as *Thymus vulgaris L.* (or thyme) or oregano. Carvacrol and thymol are generally considered safe for consumption, with medical applications in periodontal infections, and also as antibacterial additives [241]. Recently, it was reported that EOs extracted from *Thymus daenensis* and *Satureja hortensis* by hydrodistillation and analyzed by chromatography–mass spectrometry exhibited antimicrobial and antibiofilm effects against *E. coli* serotype O157:H7 (EHEC), a virulent food-borne pathogen with global spread. The authors investigated the QS inhibition potential of EOs at sub-MIC levels by inhibition of swimming and swarming motility and confirmed the effects on QS-system-related genes by RT-qPCR [242]. Mobility and chemotaxis are key factors that mediate host invasion in Salmonella serovars, and generally, for enteric pathogens, such as EPEC pathotype cells, flagellar motility is very important for the adherence to the host intestinal epithelial cells, favoring the movement across the mucus layer and the contact with host cell receptors [128].

#### 4.6.2. Polyphenols

An example of a polyphenol is epigallocatechin gallate (EGCG), which is a bioactive compound present in the fruit and leaves of plants such as *Camellia sinensis*, the source of green and black tea [243]. EGCG has previously been demonstrated to have antibacterial activity against many bacterial species [244], alone and also in combination with antibiotics and other phytochemicals against multidrug-resistant (MDR) Gram-positive and Gram-negative bacteria [245]. EGCG has a wide variety of antibacterial effects [246] and also acts as a QSI; thus, it inhibits the swarming mobility of *P. aeruginosa* PAO1 cells and downregulates the genes Las and PQS of the QS mechanism [162]. EGCG also plays an important role in binding with various proteins responsible for biofilm formation [247,248,249].

Inhibition of intercellular signaling by natural QSIs is a very attractive alternative to antibiotherapy and is an ecological strategy for fighting chronic infections and BAIs.

QSIs are secreted by all organisms, including plants, microorganisms and animals [11,13], as an expression of innate anti-infectious defense mechanisms. For instance, some insect extracts (hive products, such as propolis (a very complex product) and melittin (the main component of honeybee venom)) have shown antimicrobial and antibiofilm properties. Moreover, melittin, a natural peptide extracted from honeybee venom, in a non-toxic concentration, exhibited synergistic activity with antibiotics (doripenem and ceftazidime) combinations used against MDR isolates of *Acinetobacter baumannii* and *Pseudomonas aeruginosa* [11].

QSI treatment for BAIs seems the obvious indication, allowing a quick assessment of their in vivo efficiency [13].

Natural compounds can present some disadvantages, such as their presence in small quantities in plants and the isolation and purification methods being expensive; they sometimes raise problems of stability and availability (dependency on the vegetative cycle and development in certain climates) or do not meet the ADMET conditions (absorption, distribution, metabolism, excretion and toxicity) [250]. In addition, their antimicrobial mechanisms of action should be fully elucidated, and, at the same time, the potential synergistic effects with conventional antibiotics as a solution to combat bacterial resistance phenomena would also be worth studying [251].

In general, natural substances have some limitations due to their low availability and stability, high volatility and great diffusion capacity which do not support their current clinical use. These disadvantages lead to the necessity to develop new delivery methods for these therapeutical agents (vectorization and controlled delivery at target/infection site) to improve their efficiency. However, their great potential completely justifies the research efforts [4].

Once the efficacy of certain natural substances is proven, synthetic biology can be used to produce these substances in large amounts [252], because this strategy also has to be sustainable.

### 4.7. Combination-Based Antipathogenic Therapies

Combination-based strategies are now considered the most appropriate strategies for treating infections with MDR strains and/or biofilm-associated infections. Although the first recommended and recognized combinations of antibiotics date back a long time (1950), the number of those with a synergistic effect is limited, while the syncretic combinations of antibiotics with various adjuvant compounds are targeted more in literature nowadays [253] (some of them were already mentioned in Table 1—antibiofilm strategies).

For instance, the antibacterial activity of polyphenols such as epigallocatechin gallate (EGCG) against many bacterial species is well known [243]. However, EGCG has been demonstrated to be active alone or in combination with antibiotics and other phytochemicals against multidrug-resistant (MDR) Gram-positive and Gram-negative bacteria [244,245]. Its antibacterial activity and synergism with antibiotics are explained by different potential mechanisms such as interaction with the structure or synthesis of the bacterial cell wall, enzyme inhibition, oxidative stress, leakage of cell content and increased membrane permeability [254]. A study from 2019 [255] demonstrated that there is a synergy demonstrated in vitro and in vivo between aztreonam and EGCG against multidrug-resistant (MDR) clinical strains of *P. aeruginosa*. It is also shown that EGCG is able to restore the antibacterial activity of aztreonam in the normal concentration used for *P. aeruginosa* [255]. Thus, it is also proved that the tolerance of biofilm cells represents a transitory, reversible state [256].

Brackman and coworkers have studied the effect of some QSIs by targeting the QS system of *Pseudomonas aeruginosa* and *Burkholderia cepacia* complex organisms (using baicalin hydrate, cinnamaldehyde) or the QS system of *Staphylococcus aureus* (with hamamelitannin). The effects of these substances were evaluated by comparison with antibiotic activity, namely tobramycin for the *P. aeruginosa* and *B. cepacia* complex and clindamycin or vancomycin for *S. aureus* strains. The authors also tested the combinations of these antibiotics with QSIs, all variants being evaluated in different in vitro and in vivo biofilm models, and proved that the combination of an antibiotic and a QSI generally led to increased killing effects, by comparison with the killing activity of an antibiotic alone, but the reduced concentrations were strain- and model-dependent. These data suggested that QSIs may amplify the success of antibiotherapy by increasing the susceptibility of biofilm-embedded bacteria, as well as by possibly increasing host survival post-infection [257].

Such experiments have oriented research toward the production of hybrid antibiotics; examples are the coumarin-based antibiotic hybrids which have shown their activity against both Gram-positive and Gram-negative bacterial pathogens. These hybrid antibiotics represent a potential and promising perspective for overcoming the problem of multidrug resistance/PDR, with an increasing mortality rate. Now, the interest of researchers is focused on details concerning the structure–activity relationship which will lead to the improvement of these hybrids and their efficient clinical use [258].

As bacterial antibiotic resistance is continuously increasing and there are reports about multidrug- and pandrug-resistant strains, there is an urgent need for new antibacterials, based on different mechanisms of action than conventional antibiotics. One such alternative or complementary strategy is the use of enzybiotics; the term, first used in 2001 [259] is a combination of the words “enzyme” and “antibiotic”, denoting endolysins, their synthesis being coded by the phagic genome and classified in two groups: (a) peptidoglycan hydrolases (lysins) used as purified lysins, especially in recombinant form as antibacterial agents [194], and (b) polysaccharide depolymerases able to degrade bacterial exopolysaccharides, including biofilm matrix, slime layers, capsules or lipopolysaccharides [260]. Both enzymes can be used extracellularly, and in contrast to antibiotics, enzybiotics can also attack the metabolically latent or persister cells. These phage-derived enzymes have proved their efficiency in experimental in vivo infections caused by Gram-positive and Gram-negative bacteria; moreover, in recent years, peptidoglycan hydrolases were included in clinical trials [195].

In principle, antibiotic combination therapy presents several advantages, such as improving the effectiveness of antibacterial activity (synergism), broadening the spectrum of activity (extremely useful in infections of unknown etiology), combating polymicrobial infections, suppressing antibiotic resistance and decreasing toxic effects on the host. However, there are also a number of disadvantages that they could generate, such as the reduction in antimicrobial activity (antagonism) and the unnecessary consumption of antibiotics in the case of unregulated combinations, associated with the additional exposure of patients and the environment to antibiotics, which favors the transmission of MDR bacteria.

For infections with vancomycin-resistant Enterococcus (VRE) and those with *A. baumannii*, *P. aeruginosa* and *Enterobacterales* resistant to carbapenems, the literature mentions treatment options based on last-generation antibiotics or, in some cases, combinations of antibiotics [261]. However, the recent recommendations of the Infectious Diseases Society of America (IDSA) only mention a few combinations of antibiotics (ampicillin–sulbactam, ceftazidimex–avibactam, ceftazidime–avibactam–aztreonam, ceftolozane–tazobactam, imipenem–cilastatin–relebactam, meropenem–vaborbactam, trimethoprim–sulfamethoxazole).

*P. aeruginosa* is an ESKAPE pathogen and, at the same time, a research model for antivirulence agents. Rezzoagli et al. (2020) [262] used it as a model pathogen in a study regarding the combination therapy with four antibiotics and two antivirulence compounds in different variants. The results showed a synergistic effect for colistin–tobramycin and an antagonistic effect for ciprofloxacin–meropenem in combination with antivirulence compounds for both. On the other hand, this research revealed that selection for antibiotic resistance using antivirulence compounds could be reduced (ciprofloxacin–gallium, colistin–furanone), abrogated (meropenem–gallium, tobramycin–gallium) or reversed (tobramycin–furanone).

Recent meta-analyses have revealed unexpected results for the synergistic and well-known combination for Gram-negative bacteria between β-lactams and aminoglycosides. For example, out of 64 randomized controlled trials that looked at severe infections in patients without neutropenia (abdominal infections, UTIs, pneumonia and sepsis), 12 did not record a significant difference between monotherapy and therapy combinations in terms of all-cause mortality. Furthermore, the difference in bacteriological failure between the two therapies was not significant, while combined therapy seemed to increase clinical failure compared to beta-lactam monotherapy [263]. Moreover, nephrotoxicity seems to be more substantial in combination therapy than monotherapy, at least in the case of MDR Gram-negative bacteria infections (e.g., colistin—combination therapy versus monotherapy) [263]. Khayyat and coworkers (2021) used secnidazole as a virulence mitigator; secnidazole is a substance from the imidazole class of drugs that has a similar structure to metronidazole used to treat bacterial vaginitis and protozoal infections. The study proved that secnidazole (in sub-MIC) acts analogously to AHLs and inhibited QS, leading to the attenuation of *P. aeruginosa* pathogenesis [264].

Surprisingly, even other drugs, conceived for other purposes, proved an antipathogenic effect, such as tenoxicam. In a study performed by Askoura and coworkers (2020), this non-steroidal anti-inflammatory drug decreased the production of many virulence factors of *P. aeruginosa*, such as pyoverdin, pyocyanin, rhamnolipids, proteases, elastase and hemolysins. Moreover, qRT-PCR revealed a significant reduction in the expression of QS genes in *P. aeruginosa* cells exposed to tenoxicam compared to the control, an effect also confirmed by a mice infection model; these results encourage the use of tenoxicam in combination with antibiotics for the efficient therapy of *P. aeruginosa* infections [265].

Similarly, Saqr and coworkers (2021) investigated the antivirulence and anti-QS activities of the drug allopurinol (a urate-lowering drug) against the *P. aeruginosa* PAO1 strain. Allopurinol (in 1/10 MIC) significantly decreased the synthesis of the pigment violacein in *Chromobacterium violaceum* CV026, which is QS-controlled, and other *P. aeruginosa* QS-controlled virulence factors. Moreover, allopurinol decreased the infiltration of *P. aeruginosa* and leucocytes and reduced liver and kidney congestion in infected mice. By in silico study, it was shown that allopurinol could compete with the signaling molecules binding to the receptors LasR and RhlR. By qRT-PCR, it was proved that allopurinol has a significant downregulation effect on all tested QS-encoding genes that control virulence factor expression [266].

Recent studies highlighted the antipathogenic effects of metformin (biguanide), an antidiabetic drug used as first-line therapy for T2DM. It was proved that metformin had an in vitro effect of significantly attenuating the virulence features of *P. aeruginosa*, reducing the production of extracellular enzymes and inhibiting bacterial motility and biofilm development. It was proved that the antivirulence activity of metformin is due to its ability to interfere with QS and the expression of QS-controlled genes [267].

Another illustrative example is offered by the study of Cavalu and coworkers (2022) who performed a screening of twenty-two β-adrenoreceptor blockers for the anti-QS activities and identified *atenolol* as a promising candidate. This drug was tested for its anti-QS, antibiofilm and antivirulence activities against Gram-negative bacterial strains of *P. aeruginosa*, *Proteus mirabilis* and *Serratia marcescens*, firstly by in silico study, and thereafter the results were verified by in vitro and in vivo experiments. The authors proved the ability of atenolol to reduce the biofilm formation, virulence enzyme production and motility and finally its potential to protect infected mice. They concluded that atenolol exerts anti-QS and antivirulence activities and can be used as a complementary/adjuvant anti-infectious drug [268].

### 4.8. Vaccines and Immunotherapy

An attractive strategy as an alternative or complement to antibiotics is immunoprophylaxis by vaccination and the use of specific monoclonal antibodies (mAbs) targeting MDR and virulent strains of common pathogens.

Concerning the vaccination against adherent and biofilm-forming pathogens it is important to use bacterial cells with an adherent phenotype or purified adhesins for vaccine production. There were many attempts, but at the global level, only the pneumococcal vaccination was successful [6]. The main obstacle to obtaining efficient anti-adhesin vaccines is their antigenic variation; this obstacle can be by bypassed producing polyspecific/polyvalent vaccines (such as in the previous example, the pneumococcal vaccine) containing antigens from the most frequent strains circulating in the human community.

Another example of a polyvalent vaccine is a 24-valent one, anti-*K. pneumoniae* strains isolated from cases of bacteremia. *Klebsiella pneumoniae* is a capsulated bacterium that easily evades the immune system and is the most common carbapenem-resistant opportunistic enterobacteria causing frequent hospital-acquired infections that are difficult to treat. The previous *Klebsiella*-specific vaccine was proven to cover numerous serotypes among the 70 serologically distinct capsular types identified within this species [269], but there are also current attempts at immunotherapy targeting the capsular antigens [270].

Another immunological way to fight against MDR and virulent pathogens is immunotherapy. For example, recently, Nielsen and coworkers reported the development of a humanized bispecific monoclonal antibody (mAb) with specificity for clinical isolates of *A. baumannii*, in order to improve the avidity, efficacy and strain coverage of two previous anticapsular mAbs. They proved that such a novel therapy targeting an opportunistic pathogen with a high level of AR and which provokes difficult-to-treat infections could be efficient, the bispecific antibodies having properties superior to the original mAbs alone or combined, concerning binding affinity, opsonization and in vivo efficacy [271].

## 5. Conclusions

Antibiotic resistance and virulence are not two independent features of pathogens; sometimes they are co-located and transmitted together. Their association influences the fitness of bacterial strains and their dissemination capacity in the community. It is well known that for a manageable infection due to a certain microbial strain, the most appropriate treatment is an active antibiotic. However, due to the increasing rates of multidrug resistance and even PDR between strains of clinically important pathogens, there is an urgent need to find and test new alternative or complementary anti-infectious strategies, efficient and without the capacity to exert selective pressure. Unconventional strategies have already been discovered, and the search continues. Some of these strategies use natural active substances, including those with antimicrobial, QS inhibition, QQ and immunomodulatory properties, which are a huge reservoir of efficient drugs. These natural molecules (especially those of vegetal origin) have been empirically known and used in ethnomedicine, prophylaxis or immune stimulation, but now we have to move to the next level—the scientific one, with known active principles, used alone or in combination with antibiotics or nanocarriers, with determined dosages and mechanisms of action.

As the route of dissemination of resistance and virulence is represented by the mobile genetic elements, the emergence of new strains more resistant and/or virulent could represent an important health problem. Therefore, in the near future, we will need new antibiotics/antimicrobials, as well as antipathogenic molecules, interfering with virulence gene expression and converting a virulent strain into one less able to induce an infectious process, being more susceptible to the immune system; using this strategy, the spread of resistant strains will also be avoided.

There are also preoccupations regarding the selection of new probiotics, even archaebiotics, which are able to re-establish the eubiosis status or to increase the anti-infectious barrier effect in order to actively prevent infections. These live drugs are a matter of debate for international organizations concerning food safety and compliance with current rules in order to obtain their approval for large clinical use.

It is important to intensify the translational research in microbial ecology, with applications in the environment and human health, because, in fact, we are speaking now about the “one health” concept.

## Figures and Tables

**Table 1:** Trends in antibiofilm strategies.

Antibiofilm Strategies (Short Description)	Tested Strains	References (Selection)
Bacteriophages		
vB_AbaM_ISTD—phage isolated from Belgrade wastewaters	Nosocomial carbapenem-resistant *Acinetobacter baumannii* (6 h after biofilm treatment, not 24 h)	[46]
PM-477 (engineered lysin) phage strain— disruption of biofilm without affecting the remaining vaginal microbiome	*Gardnerella* sp. (biofilm-forming bacteria) from bacterial vaginosis patients	[47]
Recombinant tailspike protein (TSP, showed enzymatic activity) of φAB6 phage	*Acinetobacter baumannii* (inhibit biofilm formation and degrade formed biofilm)	[48]
Bacteriocins		
Bacteriocins produced by *Enterococcus faecium* (crude supernatants of non-pathogenic strains)	*Streptococcus mutans* ATCC 25175-associated preformed biofilms	[49]
Purified bacteriocin (100 μg/mL) from *L. lactis* strain CH3	*S. aureus*; *S. flexneri*; *K. pneumoniae*; *S. pyogenes*; *C. albicans*; *A. fumigatus*, 24 h biofilms	[50]
BM1300—produced by *Lactobacillus crustorum* MN047	*S. aureus; E. coli*, 24 h biofilms (crystal violet assay)	[51]
Antimicrobial peptides (AMPs)		[52]
KP and L18R (antifungal peptides)	*Enterococcus faecalis*	[53]
Pro10-1D, a potent AMP that inhibits biofilm formation, could be considered with an insect source (synthesis based on AMP structures previously isolated from a beetle defensin—*Protaetia brevitarsis*)	*E. coli*; *A. baumannii* (including MDR strains)	[54]
Temporin G (FFPVIGRILNGIL-NH_2_)—animal origin (isolated from *Rana temporaria*)	Preformed *S. aureus* biofilms	[55]
Cathelicidin peptide SMAP-29 (sheep myeloid AMP)	*Acinetobacter baumannii*	[56]
Cathelicidin-derived peptide D-11	*Klebsiella pneumoniae*	[57]
Puroindoline	*Campylobacter* spp.	[58]
Melectin, the first peptide identified in the solitary bee venom, a cationic amphipathic peptide with rich hydrophobic and basic amino acid residues and a proline	*S. aureus*; *P. aeruginosa*	[59]
Natural extracts [60]		
Plant extracts: Essential oils (EOs) and components *Clove (Eugenia caryophyllata)* EOs	*Listeria monocytogenes*, *Salmonella enteritidis*	[61]
Eucalyptus (*Eucalyptus globulus* Labillardiere), sage (*Salvia officinalis*) EOs	*P. aeruginosa* (hospital-acquired and wastewater strains), 24h biofilms	[62]
Eugenol (0.4%)	Antibiotic-resistant *Vibrio parahaemolyticus* (time-kill assay—on surface of crab shells)	[63]
Carvacrol (1.9 mM)	*P. aeruginosa* (by numbering attached cells on stainless steel and by fluorescence microscopy)	[64]
Phenolic compounds:		
Gallic acid Tannic acid	*E. coli* (*csgA* mutant biofilm) *E. coli* (*pgaA* and *recA* mutant biofilms)	[65]
2-hydroxy-4-methoxybenzaldehyde (from *Hemidesmus indicus*)	*Staphylococcus epidermidis*	[66]
Pulverulentone A (from *Callistemon citrinus*)	MSSA and MRSA	[67]
Phloretin (37.28, 74.55, or 149.10 μg/mL)	*C. albicans*, 24 h biofilms (by crystal violet assay)	[68]
12-methoxy-trans-carnosic acid and carnosol (from *Salvia officinalis* L.)	*Candida* sp.	[69]
Emodin (an anthraquinone from *Polygonum cuspidatum*)	*Candida* sp.	[70]
Quercetin, myricetin and scutellarein	Specifically inhibited *Bap*-mediated biofilm formation ^1^ of *S. aureus* and other staphylococci	[71]
Pure rutin and rutin in combination with gentamicin	MDR strains of *P. aeruginosa*	[72]
Other natural compounds		
L-homoserine lactone, ajoene, allicin (from garlic, *Allium sativum* L.)	*P. aeruginosa*	[73]
Cannabidiol	MSSA and MRSA	[74]
Bee products—compound source		
Propolis extract 27-Hydroxymangiferonic acid (from propolis extract) Ambolic acid (from propolis extract) Mangiferonic acid (from propolis extract)	*S. aureus*; *E. coli*; *P. aeruginosa*; *C. albicans*; *C. tropicalis* *S. aureus*; *L. monocytogenes*; *E. faecalis*; *E. coli*; *C. tropicalis* *S. aureus*; *E. faecalis*; *E. coli*; *S. typhi*; *C. albicans*; *C. tropicalis* *S. aureus*; *L. monocytogenes*; *E. faecalis*; *E. coli*; *S. typhi*; *C. albicans*; *C. tropicalis*	[75]
Bee pollen ethanol extracts	*S. aureus* ATCC 25,422; *P. aeruginosa* ATCC 25,853; *C. glabrata*	[76]
Melittin	Effective alone against the strong biofilm of MDR pathogens (*S. aureus*; *P. aeruginosa)*	[77]
Nanoparticles (NPs)		
NPs—antimicrobial, antibiofilm, antipathogenic	Bacteria (including MDR strains), microfungi protozoa, viruses	[78]
Hordenine-AuNPs	*P. aeruginosa* PAO1	[79]
AuNP *Capsicum annuum* extract	*Pseudomonas aeruginosa* PAO1 and *Serratia marcescens* MTCC 97	[80]
Silver nanoparticles (AgNPs)	MDR strains of *Acinetobacter baumannii*, 24 h biofilms (by crystal violet assay)	[81]
AgSiO2 nanoparticles	*Staphylococcus aureus*	[82]
Mesoporous Fe_3_O_4_@SiO_2_ NPs containing glucose-oxidase and l-arginine produce NO by a cascade reaction	vancomycin-resistant *S. aureus* biofilms in vivo (in mice)	[83]
A magnetic microswarm based on porous nanocatalysts (mesoporous Fe_3_O_4_) could eliminate biofilms by generating ROS ^2^	*E. coli*; *B. cereus*	[84]
Nanobioactive surface based on magnetite@eugenol and (3-hidroxybutyric acid-co-3-hidroxyvaleric acid)–polyvinyl alcohol microspheres	*Staphylococcus aureus*; *Pseudomonas aeruginosa*	[85]
Chrysin-loaded chitosan NPs	*Staphylococcus aureus*	[86]
Zingerone-loaded chitosan NPs	*Pseudomonas aeruginosa*	[87]
Curcumin-loaded chitosan NPs	*Staphylococcus aureus*; *Candida albicans*	[88]
NP-based dissolving microneedles with doxycycline	*Staphylococcus aureus*; *Pseudomonas aeruginosa*	[89]
Lipid-based NPs: monoolein with tobramycin	*Pseudomonas aeruginosa* (cystic fibrosis-related)	[90]
Physical modern methods based on light or ultrasound for biofilm removal		
Multisonic/ultrasonic protocols with various applications in restorative dentistry and endodontics	Multispecies biofilm removal	[91,92]
Antibiofilm photodynamic therapy	Vancomycin-resistant *Staphylococcus aureus* (VRSA)—induced infection (in vitro and in vivo)	[93]
Biological methods based on interspecific antagonism		
Competition/probiotics		
Lactobacilli-derived exopolysaccharides (*Lactobacillus crispatus* and *Lactobacillus gasseri*)	Stimulating the biofilm formation of lactobacilli and preventing, at the same time, the biofilm formation of *Escherichia coli*; *Staphylococcus* spp.; *Enterococcus* spp.; *Streptococcus agalactiae*; *Candida* spp.	[94]
*Bifidobacterium lactis* and *Bifidobacterium infantis*—antagonist effect (alone or in combination)	*Porphyromonas gingivalis; Fusobacterium nucleatum*	[95]
Probiotic yeast *Saccharomyces boulardii* CNCM I-745 through modification of the extracellular matrix composition	*Clostridioides difficile* biofilm formation (in vitro)	[96]
Predatorism *Bdellovibrio* and similar organisms (with an obligatory predatory lifestyle) could be from *Micavibrio* genus (a-proteobacteria) or *Bdellovibrionaceae*, *Bacteriovoraceae*, *Peredibacteraceae*, *Halobacteriovoraceae* and *Pseudobacteriovoracaceae* family (d-proteobacteria)	Exclusive predator of Gram-negative Gram-positive biofilms also seem to be prone to degradation Alters the ecology of biofilm assembly when new cells, non-predatory, enter the system (*E. coli* was able to penetrate *V. cholerae* biofilms exposed to predators)	[97,98]
*Bdellovibrio bacteriovorus*	*Pseudomonas aeruginosa* or *Staphylococcus aureus* (collected from sputa of two cystic fibrosis patients) biofilms	[99]
New synthetic chemical compounds		
Small organic molecules—derivatives of the following: Imidazoles; 2-aminoimidazoles/triazoles; Pyrazoles; Indole and carbazoles; 2-phenylhydrazineylidenes; Pyrroles; Phenazines and quinolines; Cynnamides.	*P. aeruginosa* MRSA; *A. baumannii*; *V. cholera*; *P. aeruginosa* *S. aureus*; *S. epidermidis* *E. coli O157:H7*; *P. aeruginosa* *S. aureus* *S. aureus*; *S. epidermidis* *S. aureus*; *E. faecium*; *S. epidermidis* *P. aeruginosa*	[100]
Metal compounds with Ga (III)—simple salts or complexes Gallium Meso- and Protoporphyrin IX	*P. aeruginosa* biofilm formation in vitro and in murine lung infection models Biofilms of MDR strains of *Acinetobacter baumannii*	[101]
FN075 (with ring-fused 2-pyridones) blocks biogenesis of curli and type 1 pili and presents unique antibiofilm properties	Uropathogenic *Escherichia coli* (UPEC)	[34]
Combined drug therapy		
Vitexin (flavonoid) with azithromycin and gentamicin		[102]
Curcumin treatment followed by light irradiation (10 J/cm^2^)—photodynamic therapy	*Pseudomonas aeruginosa*	[103]
Gentamicin (GEN), vancomycin (VAN), tetracycline (TET), ciprofloxacin (CIP), daptomycin (DAP), erythromycin (ERM) and linezolid (LIN) showed a significantly increased efficacy at 2× MIC against phage-treated biofilms compared with intact biofilms	*S. aureus* Newman (72 h biofilm)	[104]
PlySs2 (phage) and vancomycin reduced (92%) the number of CFUs on the implant surface (when used together) in vivo	*S. aureus* (murine tibial implant)	[105]
Depolymerase Dpo71 (from a bacteriophage specific for *Acinetobacter baumannii*) alone and in combination with colistin	Inhibits formation and disrupts preformed biofilms; combination enhances the antibiofilm activity and improves the survival rate of *Galleria mellonella* (infected with *A. baumannii*)	[106]
Melittin synergism with doripenem and ceftazidime (as topical drug) Melittin synergism with gentamicin, ciprofloxacin, vancomycin, and rifampin	*Acinetobacter baumannii*; *Pseudomonas aeruginosa* Effective against the strong biofilm of MDR pathogens (*S. aureus* and *P. aeruginosa*)	[11,77]
Tetrasodium EDTA, ethanol and chlorhexidine hydrochloride	*S. aureus*; *S. epidermidis*; *P. aeruginosa*; *P. mirabilis*; *E. coli* (MBEC Assay)	[107]
Encapsulation of eugenol and triclosan into polymeric nanoemulsions acts synergistically.	Murine model of mature MDR wound biofilm infections (in vivo)	[108]
TB_KKG6A and TB_L1FK (AMPs) and EDTA	*Pseudomonas aeruginosa*; *Staphylococcus aureus*	[109]
Monoclonal antibodies target PNAG ^3^ and Aap ^4^	*S. aureus* biofilms	[110]
Chelating agents		
Ethylenediaminetetraacetic acid (EDTA)	Prevent the biofilm formation by *Listeria monocytogenes* and *Staphylococcus epidermidis* strains; kill *Pseudomonas aeruginosa* biofilm-embedded cells	[111]

^1^ Biofilm-associated protein (Bap): scaffolds of the biofilm matrix with amyloid-like structures. ^2^ ROS—reactive oxygen species. ^3^ PNAG—poly-N-acetylglucosamine. ^4^ Aap—accumulation-associated protein.

**Table 2:** New antimicrobials from natural microbiota sources.

Taxonomic Affiliation	Producer Microorganisms	New Antimicrobial Metabolites	References (Selection)
Soil Microbiota			
Bacteria	*Bacillus subtilis group*	Ribosomal peptides, volatile compounds, polyketides (PKs), nonribosomal peptides (NRPs), and hybrids between PKs and NRPs	[133]
	*Streptomyces* spp.	Main metabolites: phenolic compounds and benzeneacetic acid. Other compounds: 1-nonadecene, nalidixic acid, a pyrrolizidine, etc., antimicrobial activity against *S. aureus* MTCC * 96 and *E. coli* MTCC 40	[134]
	*Streptomyces diastaticus subsp. ardesiacus* strain YIM PH20246	New phenazine metabolites: 6-hydroxyphenazine-1-carboxamide and methyl 6-carbamoylphenazine-1-carboxylate—antimicrobial activity against *Staphylococcus aureus* (ATCC 25923) and *Staphylococcus albus* (ATCC 10231)	[135]
	*Streptomyces* sp. strain FR7	Polyketides (with methylsalicylic acid component)—antimicrobial activity against *Micrococcus luteus*, *S. aureus*, *L. monocytogenes* and *P. aeruginosa*	[136]
	*Streptomyces chrestomyceticus* ADP4	Phenyl 2′α, 2′β, 6′β-trimethyl cyclohexyl ketone and phenyl nonanyl ether—inhibiting biofilm formed by *C. albicans* ATCC 10231	[137]
	*Streptomyces* sp. Je 1–651	spiramycins, stambomycins and unidentified compounds—antimicrobial activity against many bacteria and yeast strains	[138]
	*Streptomyces agglomeratus* 5-1-3	Nonribosomal peptide echinomycin showed excellent anti-MRSA activity	[139]
	*Streptomyces spectabilis*	Metacycloprodigiosin—antimicrobial activity against eight clinically common pathogens: *S. aureus*, *B. subtilis*, *E. coli*, *S. pyogenes*, *P. aeruginosa*, *B. typhi*, *C. albicans* and *Trichophyton rubrum*	[140]
	*Streptomyces* sp. KIB-H1318	Three new phenoxazinone-related alkaloids—minor antibacterial activity against *E. coli* ATCC 8099, *B. subtilis* ATCC 6633 and *S. aureus* ATCC 6538	[141]
	*Streptomyces* sp. (ERINLG-201)	Bluemomycin, a new naphthoquinone derivative—antimicrobial properties against Gram-negative bacteria	[142]
	*Streptomyces* sp. (CL12-4)	New bicyclic diterpenoid, benditerpenoic acid—exhibits moderate antibacterial activity against methicillin- and multidrug-resistant *S. aureus.*	[143]
Fungi	*Penicillium herquei* MA-370	α-pyrone derivatives—antimicrobial activity against *P. aeruginosa* and *E. coli*	[144]
Human Normal Microbiota			
Bacteria	*Staphylococcus lugdunensis* (nasal microbiota)	Lugdunin (nonribosomally synthesized cyclic peptide)	[145,146]
	*L. gasseri* 1A-TV, *L. fermentum* 18A-TV, and *L. crispatus* 35A-TV (vaginal microbiota)	Metabolites produced by cell-free supernatants (CFSs) and their combination—strong bactericidal effect on the tested multidrug-resistant urogenital pathogens (*S. agalactiae*, *E. coli*, *KPC-producing K. pneumoniae*, *S. aureus*, *P. aeruginosa*, *P. mirabilis*, *P. vulgaris*)	[147]
	*Coagulase-negative Staphylococcus (CoNS)* including *S. epidermidis* and *S. taphylococcus hominis isolates* (healthy skin)	Antimicrobial peptides (AMPs)—antibacterial activity against *S. aureus*	[148]
	Gut microbiota	The microbial metabolites include amino acids, nonribosomal peptides and ribosomally synthesized post-translationally modified peptides, lipids, glycolipids, oligosaccharides, terpenoids, polyketides and others—antimicrobial activities	[149,150]
Archaea	*Methanobrevibacter smithii* (*intestinal microbiota*)	Archaebiotics (probiotics of archaeal origin)—potential antagonists against gut pathogens	[149]
Deep Sea Water Microbiota			
Bacteria	*Bacillus pumilus*	Pumilacidin active against *Staphylococcus aureus*	[151]
	*Streptomyces* sp. ZZ745 (isolated from marine mud, collected from a coastal area)	Bagremycins F and G showed antibacterial activity against *Escherichia coli*	[152]
Fungi	New *Micromonospora* strain, designated 28ISP2-46T, recovered from the microbiome of a mid-Atlantic deep-sea sponge	Kosinostatin and isoquinocycline B (isolated from 28ISP2-46T fermentation broths) exhibit antibiotic properties against many MDR clinical isolates	[153]

* Microbial Type Culture Collection and Gene Bank (MTCC).
